# Quality of Life of Patients Using Esophageal Speech after Total Laryngectomy: A Systematic Review Study

**DOI:** 10.3390/jpm14080817

**Published:** 2024-07-31

**Authors:** Panagiotis Plotas, Stylianos N. Mastronikolis, Angelos Papadopoulos, Kiriaki Zarnomitrou, Marina Pagkalou, Anastasios Kantanis, Eleni Alexiou, Eygenia Katseri, Maria Kyriakopoulou, Maria Reppa, Aggeliki Souka, Alexandros Christopoulos, Nikolaos Trimmis, Nicholas Mastronikolis

**Affiliations:** 1 Laboratory of Primary Health Care, School of Health Rehabilitation Sciences, University of Patras, 26504 Patras, Greece; up1096242@ac.upatras.gr (S.N.M.); up1105395@ac.upatras.gr (M.P.); up1047795@upnet.gr (A.K.); nicktrimmis@upatras.gr (N.T.); 2 Department of Speech and Language Therapy, School of Health Rehabilitation Sciences, University of Patras, 26504 Patras, Greece; up1110303@ac.upatras.gr (A.P.); up1076042@ac.upatras.gr (K.Z.); up1076089@ac.upatras.gr (E.A.); upw800274@ac.upatras.gr (E.K.); upw800358@ac.upatras.gr (M.K.); up1080000@ac.upatras.gr (M.R.); up1076090@ac.upatras.gr (A.S.); up1076098@ac.upatras.gr (A.C.); 3 General Children’s Hospital of Patras “Karamandaneio”, 26331 Patras, Greece; 4 Department of Otorhinolaryngology—Head and Neck Surgery, School of Medicine, University of Patras, 26504 Rion, Greece

**Keywords:** systematic review, quality of life, laryngectomy, esophageal speech

## Abstract

(1) Background: The present systematic study aimed to assess whether using esophageal speech (ES) as a method of vocal rehabilitation in patients after total laryngectomy enhances their quality of life (QoL) and vocal functionality based on patients’ reports. (2) Methods: Data collection was conducted from PubMed, Google Scholar, and Speech Bite, and the PRISMA Flow Diagram tool was used to record different stages of the literature search process. In the review, nine studies were included, while a bias check was carried out using the Critical Appraisal Skills Programme (CASP) checklists. Survey analysis incorporated quantitative and qualitative data, including standardized questionnaires and audio analyses. (3) Results: A technique’s effectiveness depends on the method’s functionality and the patient’s abilities. Furthermore, the findings revealed that ES use unexpectedly affects quality of life regarding patients. While statistical analysis of the studies showed that some patients reported improvement in quality of life and vocal functionality, others faced challenges such as difficulty in learning the technique, long-term intervention, and unsatisfactory phonetic performance. Some studies observed quantitative measures, such as improved Voice Impairment Index (VHI) scores and Voice-Related Quality of Life (V-RQOL) scores. However, results were not uniformly positive across studies, with a subset of patients reporting minimal improvement. (4) Conclusions: The limited literature on the effect of ES on patients’ QoL appears to influence the results in different ways. However, research data support that patients’ communication and psychological state seem to improve significantly compared to patients who have not been rehabilitated. The final assessment of the technique’s effectiveness on quality of life must depend on many factors.

## 1. Introduction

Laryngeal cancer is estimated to account for approximately 25% to 30% of head and neck malignancies [1,2,3] while representing only 2% of all malignancies [4]. In 2019, a study [5] found that about 130,000 new cases are diagnosed annually and that there is an average of 80,000 deaths annually caused by this type of cancer. Additionally, it occurs more often in men (6.17 cases/100,000) than in women (1.20 cases/100,000) [1]. Considering the data mentioned above, laryngeal cancer is the most common form of carcinoma in the area of the upper respiratory tract [6]. The cancer is at an advanced stage in a large percentage of these cases, and therefore, patients undergo a total laryngectomy (TL). This is an interventional method aimed at eliminating malignant neoplasms. However, they may affect the larynx at a functional level [6].

The larynx controls and determines three main functions: (a) breathing, (b) swallowing, and (c) voicing. After TL surgery, these three functions change radically and significantly affect patients’ quality of life (QoL). Impaired speech production and intelligibility, for example, impair QoL. Human health is defined by the absence of disease and complete physical, mental, and social well-being [7]. The loss of natural voice and limited speech intelligibility of patients after laryngectomy negatively affect their QoL, resulting in social stigma and psychological burden [5]. According to the World Health Organization [8], QoL is defined as “the individual’s perception of his place in life, in the context of the culture and systematic values in which he lives and in relation to his goals, expectations, standards, and concerns.” Although the speech disorder is not the only factor that affects the QoL of patients with total laryngectomy, it nevertheless has a primary influence on the social relationships of individuals, increasing the feeling of loneliness and social isolation [5].

Promoting the patient’s quality of life and offering vocal health and functionality are the main goals of voice rehabilitation. The choice of each technique is a very factorial process that requires the patient’s cooperation with the multidisciplinary team. Considering the patient’s unique needs and abilities, we consider many factors before making the final decision [9]. The patient’s medical history, demographics, quality of life, and psychological support are some of the factors that will shape the decision. There are three appropriate methods of voice restoration after total laryngectomy: (a) tracheoesophageal speech, (b), electrolaryngeal voice, and (c) esophageal speech [10].

During the tracheoesophageal speech, the air is directed into the esophagus, where it is then expelled, causing vibrations in the mucous membrane of the pharyngoesophageal segment. The technique allows for a more natural voice and has demonstrated a general improvement in the quality of life of patients. However, it is a maintenance technique for vocal prostheses and has several complications. On the other hand, during electrolaryngeal speech, the restoration is performed externally, i.e., through the electrolaryngeal device [11], which is placed on the throat or cheek to produce sound [8]. The technique is relatively easy to learn but the voice is often robotic or monotone. In addition, the device is transparent in use, which affects the patient’s self-esteem and social interactions [12]. Esophageal speech is considered a relatively common method of restoration that does not require any device. The process of phonation is achieved through the pharynx and esophagus. In more detail, speech is carried out by injecting air into the esophagus, which quickly exits, creating vibration and producing speech. ES is a complex technique to learn to speak, as only 60% of patients can master it [12]. Nevertheless, the voice is produced more naturally through ES compared to the other two methods and does not need special maintenance, significantly improving patients’ QoL.

The best rehabilitation technique for people with total laryngectomy has not yet been established, but it has been found that better voice quality improves patients’ quality of life [8]. Effective voice rehabilitation can significantly affect patients’ QoL, as improved speech abilities can affect their psycho-emotional domains [13]. The evaluation of the effectiveness of therapeutic techniques needs to be multidimensional, and the choice of an altered speech, such as esophageal speech, can be assessed through auditory analyses, perceptual assessments, and patient self-reported outcomes [10]. Acoustic analysis assesses pitch and width measurements of speech but is only sometimes sufficient to measure this type of alternative speech.

On the other hand, there is a great need for detailed perceptual assessments but also for the use of QoL assessment tools to provide more structured feedback on patients’ rehabilitation experiences. Perceptual evaluations of modified voice require approaches that will be very well thought out and detailed because there appear to be several deviations of modified speech compared to standard laryngeal speech [10]. As a result, the evaluation of surrogate voices is preferably conducted through an overall impression of voice quality and speech intelligibility. This is conducted from the patient’s perspective through quality of life assessment tools [10]. These tools are important for assessing the impact of rehabilitation on patients’ lives and providing feedback on patients’ rehabilitation experiences in a more structured way [1]. Nevertheless, it must be mentioned that there is no ideal measure for evaluating the QoL because it consists of many objective and subjective characteristics. Quality of life questionnaires are the most common, and their use is widespread due to their ease of application, replication, and validation in different populations [4].

Several tools are available to assess the QoL of head and neck cancer patients. For example, there is the Voice Handicap Index (VHI) [14] that has been validated and used recently in many languages [15,16,17,18] and the Voice-related Quality of Life (V-RQOL) [19], which also has been validated in many languages [16,20,21,22] and provides detailed assessments of speech rehabilitation outcomes [10]. They can be used to assess people with dysphonia of different etiologies, including patients who have undergone total laryngectomy. They aim to investigate vocal deficits, QoL, and voice and how patients deal with specific vocal disorders due to total laryngectomy [23]. Also, the European Organization for Research and Treatment of Cancer (EORTC) questionnaires include the head and neck cancer section of the 35-item version (EORTC QLQ H&N35) and the EORTC QLQ-C30 questionnaire [24]. These tools assess both general and specific domains of patients’ lives and contain questions about speech function [10]. Finally, the University of Washington Quality of Life (UW-QOL) questionnaire [25] assesses the QoL of patients with head and neck cancer and provides information on patients’ perceptions of the most effective type of treatment [26]. Since 1993, the UW-QOL has undergone three main revisions [27]. In addition to general health-related QoL questions, it allows patients to comment on treatments through responses to open-ended questions [1].

The present study aims to determine how the use of esophageal speech affects the QoL in patients who have undergone laryngectomy due to malignant cervical cancer. The aim is to collect the best available data on the effect of the technique on patients’ quality of life and how the research results will help in making clinical decisions regarding the use or not of the above technique. This particular technique was studied to enhance and promote knowledge about it. In particular, the existence of the old literature, the limited use of E.S. after the end of the rehabilitation time, and the limited literature are some of the reasons that stimulated the group’s scientific interest. For this reason, there is a great need to update and expand the existing literature considering the limited current research on esophageal speech. The aim is to inform clinical practice and enhance the rehabilitation process, considering the quality of life of the patients in whom the intervention is provided. The reader reading this review is informed about the technique of E.S., the testimonies of patients from nine different modern types of research, and a generalized picture is created of the use of the technique. The choice of applying the technique to a patient results from many factors, and it is impossible to draw a general conclusion regarding the exclusion or non-determination of a particular group of patients. More specifically, laryngectomies are a suitable application group for E.S. However, psycho-emotional, social, and medical factors will influence the final decision to use it. In the study’s methodology, audio analyses and questionnaires were examined to determine the effect of this speech rehabilitation method on the QoL. In the continuation of the review, the results of the above evaluations are presented, as well as the conclusions regarding the QoL of laryngectomized patients after using esophageal speech.

## 2. Materials and Methods

The research was carried out in the form of a systematic review. The researchers looked for studies on the QoL of patients who use esophageal speech after laryngectomy for cervical cancer. The search for studies started in March 2023 and ended in May 2023. The studies searched were systematic reviews/meta-analyses, randomized controlled trials, cohort studies, retrospective studies, and case or case series studies. The studies searched used a mixed methodology incorporating both quantitative and qualitative approaches to the impact of esophageal speech on patients’ quality of life. The quantitative component of the studies included standardized questionnaires and acoustic analyses, while the qualitative component included semi-structured interviews to collect the experiences of the patients participating in the study. Finally, the written language of the searched studies was English, while the research review was expressed in Greek and English. The reporting of this systematic review was guided by the standards of the Preferred Reporting Items for Systematic Review and Meta-Analysis (PRISMA) Statement [28,29]. The study has not been registered in protocol.

### 2.1. Inclusion Criteria

In order to be included in the analysis, studies had to meet the inclusion criteria:

- Studies had to have been published between 2010 and 2023. The limitation was set to highlight contemporary findings related to the subject.
- The study population should be laryngectomized patients, male or female, over 45 years of age, with malignant primary cervical cancer. The specific age threshold serves to avoid hereditary cancer, and the high quality of life takes into account the socio-economic autonomy of the patients [30,31].
- The voice therapy that patients have received should have been performed using the esophageal speech technique.
- Studies should include questionnaires and auditory analyses to assess patients’ QoL after using esophageal speech. Failure to comply with the above is a criterion for exclusion from the research analysis.

### 2.2. Data Sources and Search Strategy

Searchers create a PICO-style question to search databases [32]. Regarding data collection, a search was carried out using the following databases and search engines: PubMed, Google Scholar, and Speech Bite. Additionally, the following keywords were used for the search: (quality of life) AND (total laryngectomy) OR (laryngectomy) AND (esophageal speech) AND (laryngeal cancer). The search for the studies considered suitable to be included in the research was conducted according to the selection criteria, which were predetermined after consultation and a meeting with all the researchers. These criteria were strictly defined by the needs of the research, and compliance with them, in the first phase in a more general context, was a necessary condition for all the articles that the researchers would propose. Equally important were the keywords to be used, which were pre-defined similarly. Finally, the search results in the databases were filtered by the publication date of issues and/or publications from 1 January 2010 to the period in which the present research was conducted. All of the aforementioned ensured the safe possible repetition of the search by the researchers, should this be deemed desirable.

### 2.3. Article Selection and Data Collection Process

The articles were collected from databases (*n* = 42) and reviewed for clarity based on the title and abstracts, followed by a full-text review. They were then distributed to all researchers who summarized the data and applied strict selection criteria to successfully select the most appropriate articles for this research. Studies that did not meet the above inclusion criteria or the type of research sought by the researchers were excluded from the study. The screening of each article/study and the documentation of its effectiveness in successfully collecting data suitable for the review were conducted step by step. At this point, only the selected articles were distributed to the researchers. Each researcher was asked to review the articles that were eventually included in the study (*n* = 9) and to thoroughly review the full texts. The highlighted parts were as follows:

- The aim of the study.
- The participants.
- The procedures that were followed and/or the tools that were used.
- The results of the study and the conclusions drawn by the authors.
- Information on bias control.

After collecting and reviewing all studies, the relevance of the data to the present review was evaluated. Data were collected on the patients’ QoL, their general functionality, communication functionality, voice quality, and the emotional effect of using esophageal speech. No disagreement arose between the researchers during data collection and analysis. Finally, it was deemed necessary by the researchers to analyze the central trends of the literature, in order to discover the characteristics and general trends that affect the specific research field. The entire process, from the identification to the studies’ final selection, is described diagrammatically below (Flow Diagram).

### 2.4. Participant Characteristics and Sample Size

Demographic information such as the age and gender of the participants was extracted from each study included in the survey. They included men and women above 45 years of age. The sample size of the studies studied varied, including 8 studies with small sample sizes (*n* < 50 patients), 2 studies with medium sample sizes (50 ≤ *n* < 100 patients), and 3 studies with large sample sizes (100 ≥ *n* patients). Some studies included the sample and control groups of healthy individuals or patients who had received partial laryngectomy or other treatments for laryngeal cancer.

### 2.5. Bias Check

**Figure 1:**
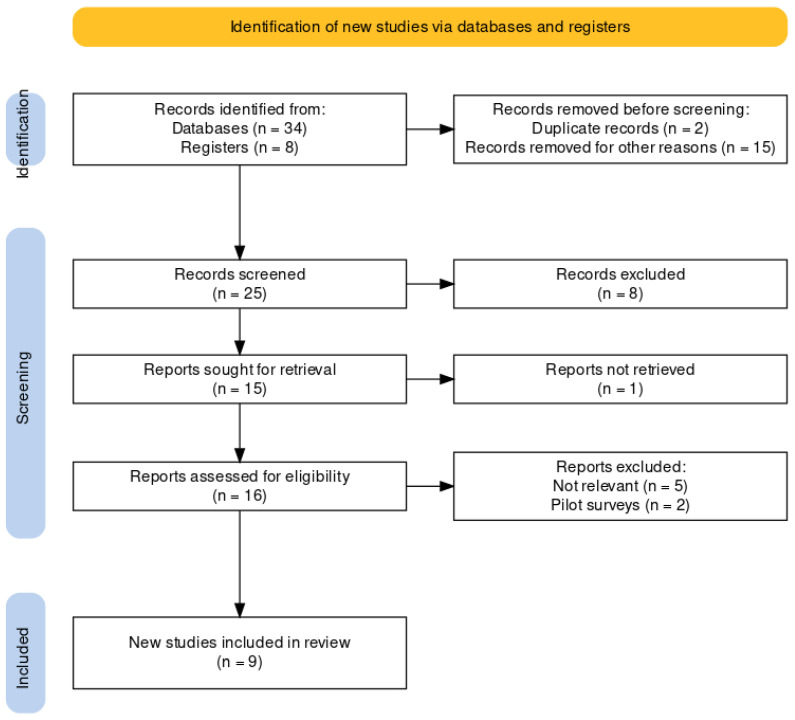
Flow Diagram (PRISMA, 2020): study identification and selection process.

Critical Appraisal Skills Programme (CASP) checklists [33] were used to assess the quality of individual studies critically (Table 1a,b). Specifically, CASP checklists were administered separately to each study to assess their reliability, clarity, and effectiveness [33]. Evaluating the reliability and quality of the studies is important for utilizing the correct information and synthesizing the data. Studies with risks of bias were excluded from the survey because the quality and reliability of their results were questionable. Figure 1 shows the Flow Diagram (PRISMA, 2020) and study identification and selection process [34].

## 3. Results

Most of the studies included in this review utilized a quantitative research design with a longitudinal approach. Standardized questionnaires were used, which assessed various aspects of patients’ QoL, voice function, and rehabilitation outcomes. After analyzing the relevant articles, it is essential that they all have a common research methodology to evaluate the QoL of patients who have undergone total laryngectomy and the effect of different rehabilitation methods on their QoL. The main questionnaires used in the surveys were the following:

- Voice Handicap Index (VHI)—in five studies.
- Voice-Related Quality of Life (V-RQOL)—in two studies.
- Short-Form Health Survey (SF-36)—in one study.
- European Organization for Research and Treatment of Cancer Quality of Life Questionnaire (EORTC QLQ-C30)—in two studies.
- HEAD & NECK CANCER (EORTC QLQ-H&N35)—in two studies.
- University of Washington Quality of Life (UW-QOL)—in two studies.
- Functional Assessment of Cancer Therapy-General (FACT-G)—in two studies.
- Groningen Enjoyment of Speech Questionnaire (GESQ)—in two studies.
- Hospital Anxiety and Depression Scale (HADS)—in one study.
- Portuguese Self Evaluation of Communication Experiences after Laryngectomy Cancer Questionnaire (P-SECEL)—in one study

### 3.1. Analysis of the Studies

The analysis of the studies in the present review revealed the impact of ES on laryngectomized patients’ lives (Table 2). There is a consensus among the studies regarding the effectiveness of the method. However, there are also opposing claims. As mentioned above, research on the effectiveness of using ES and its positive or negative effect on patients’ QoL is varied and shows a multitude of conclusions. Our research has confirmed that ES is a technique for which there are no clear conclusions regarding the degree and level of enhancement it offers overall to the patient. Four research [4,6,36,37] reports’ results support the positive effect of ES on patients’ quality of life but not on overall functionality. In particular, an enhancement in the level of well-being was observed, a fact supported by the statistical analyses of the studies. In addition, this technique is preferred over the others due to its non-invasive nature, as it does not require surgical intervention, and its cost is significantly low [4].

However, most studies (*n* = 5) report negative data regarding the improvement in the QoL. According to self-report questionnaires, patients reported that it was challenging to learn the technique and that the long-term intervention was a disadvantage. Long-term management appears problematic for laryngectomized people, resulting in a negative image of their vocal skills when using esophageal speech [40]. In addition, numerous studies support that using esophageal speech requires long-term training and intensive but conservative treatment to be applied effectively and satisfactorily [41]. Voice quality is of significant concern to patients, and they attach great importance to its improvement. Using esophageal speech does not enhance vocal performance/fluency, limiting the QoL positively. Moreover, studies described the emotional aspect of voice-quality questionnaires [4,5,6,35,37,39]. The results suggested that users of esophageal speech present a strong feeling of fear and anxiety. The levels of self-confidence and self-esteem are also low, with the social–emotional functionality appearing limited overall [1,4,5,10,40]. In a single study [30], no statistically significant effect was found related to the influence of the disorder on the emotional aspect. Using esophageal speech reduced patients’ effective communication, with three studies reporting reduced overall functionality [1,5,10,35].

### 3.2. Studies’ Findings

Studies presented consistent findings regarding the impact of voice rehabilitation on QoL, as patients who underwent voice rehabilitation with either esophageal or tracheoesophageal speech showed significant improvements in their overall QoL and communication compared to those who did not undergo rehabilitation. The overall results from most studies indicated that tracheoesophageal speech may be a more effective and preferable method of voice restoration than esophageal speech. SF-36, VHI, and self-reported quality of life statements of patients during ES implementation appear worse than TEP patients. The patients’ self-reported statements about the quality of life during the ES application seem worse than the TEP patients. Patients report an unsatisfactory experience using the technique, focusing mainly on the social–emotional level. This demonstrates that the esophageal speech method is not the one with the best results in patients who have undergone total laryngectomy and has a controversially significant impact on patients’ quality of life and overall functionality.

### 3.3. Bias Sources

Regarding the quality and potential sources of bias in studies, it is crucial to ensure their findings’ validity and reliability. It is important to note that even studies with stronger methodology may have limitations, while those with weaker methodology may still include important findings. Therefore, it is important to consider the advantages and limitations of each study when interpreting the data and applying them to clinical practice. The studies are classified as follows:

- Group 1: Studies with solid methodology and a low rate of bias
  1. Quality of Life in Patients Submitted to Total Laryngectomy [4].
  2. Voice prosthesis rehabilitation after total laryngectomy: are satisfaction and QoL maintained over time? [5].
- Group 2: Studies with weak methodology and higher rate of bias
  1. Rehabilitation following total laryngectomy: Oncologic, functional, socio-occupational, and psychological aspects [37].
  2. Verbal performance of total laryngectomized patients rehabilitated with esophageal speech and tracheoesophageal speech: impacts on patient quality of life [6].
  3. Quality of life after total laryngectomy: impact of different vocal rehabilitation methods in a middle-income country [1].
  4. Speech rehabilitation during the first year after total laryngectomy [36].
  5. Voice-Related Quality of Life in Post-Laryngectomy Rehabilitation: Tracheoesophageal Fistula’s Wellness [35].
  6. Satisfaction and Quality of Life in Laryngectomees after Voice Prosthesis Rehabilitation [39].
  7. Comparison of Voice Handicap Index in Patients with Esophageal and Tracheoesophageal Speech after Total Laryngectomy.

## 4. Discussion

In conclusion, does using esophageal speech as a method of vocal rehabilitation in laryngectomized patients enhance their QoL and overall functionality? The present study compared the positive and negative aspects of using esophageal speech, reaching structured conclusions. The final results arose from a targeted and systematic search, analysis, and interpretation of data conducted under the code of research ethics. The in-depth study of the subject highlighted the importance of rehabilitation techniques aiming to strengthen patients and provide them with a good qualitative life. Rehabilitation methods are individualized according to the patient’s unique needs, and the success rate of each technique is affected differently. Speech rehabilitation is vital for laryngectomized patients, and numerous criteria will influence the choice of the appropriate technique. Loss of speech, a disorder inextricably linked to laryngectomy, is a condition of communicative exclusion and automatically limits and weakens abilities of social mediation, mental and emotional stability, and reciprocity. It is, therefore, understood that clinical research on finding the optimal speech rehabilitation technique is a major issue.

Although our research has some limitations, such as the limited contemporary literature, we collected a sufficient sample of data that informs us about the effect of ES on QoL. The studies included in the review do not report on the treatment course of ES patients, such as effective use or technique change. Data come from specific time intervals where patients completed qualitative questionnaires and underwent assessment procedures.

Regarding the use of esophageal speech, the findings of this systematic review highlight the complex nature of esophageal speech as a method of vocal rehabilitation for patients who have undergone total laryngectomy. While the efficacy of the intervention is important, it affects quality of life and vocal function in important ways. Patients’ self-reported statements relate to concerns about using the technique, although there is evidence of efficacy regarding speech system functionality. The long-term intervention, learning difficulty, and the final voice quality are the main causes of the final self-report. Our study clarifies that patients experience an emotional and mental burden by using esophageal speech, a fact that has a catalytic effect on their QoL. Even though the studies in the review presented varying results regarding the association of esophageal speech and QoL, all studies agreed to the conclusion of limited QoL, which is consistent with our study. However, it is important to emphasize that, although in most studies, esophageal speech does not have the desired patient outcome in terms of quality of life and voice-related quality of life, its application is useful and effective in many cases in the absence of some voice rehabilitation.

The study’s outcomes focus on the consistent findings about the influence of voice rehabilitation on Quality of Life (QoL). Patients who underwent voice rehabilitation with esophageal or tracheoesophageal speech showed significant improvements in communication and overall QoL compared to patients who did not undergo voice rehabilitation. Specifically, the effect of E.S. in QoL will depend on various factors related to the patient’s medical condition and psychological state. Determining the degree of effectiveness with a positive or negative bias cannot be irrevocably determined due to the unique management of each patient. Esophageal speech is an important method of voice restoration in laryngectomy patients, and the choice of its application is a combination of factors. Learning the technique is a multifactorial process and will be influenced by both the patients’ abilities/desires and the perspective of the multidisciplinary management team. Promoting the individualized rehabilitation model, we consider it important to have different intervention methods for laryngectomy so that there is room for adaptation.

Comparing the results of the present review, they seem to be in line with the results of similar studies. A study by Souza et al., (2020) [1] examines the quality of life in laryngectomy patients and finds that vocal rehabilitation, including ES, significantly affects patients’ psychosocial well-being and communication skills. These results are related to our review, which reports a high risk of limited quality of life for patients both on the psychosocial level and in overall functioning. Challenges such as the multi-complexity of learning ES and the variable effects of its vocal performance remain important barriers to the cautious use of the technique.

### Limitations

Despite the fact that studies with a low index of bias and robust methodology were included in the research, most available studies in the literature had an increased risk of bias and weaker methodology, which significantly limits the outcome of the research as well as the validity and reliability of the results obtained from the analysis of the included studies. Also, several of the included studies had small sample sizes, thus limiting the generalizability of the results. The included studies did not have long-term follow-up, which is crucial for evaluating the prolonged effect of ES on patients’ quality of life. Furthermore, the nature of the subjective assessment of quality of life is in itself a limitation that may lead to bias. A significant limitation was that no studies were entirely related to the QoL of patients after total laryngectomy using esophageal speech as a method of voice rehabilitation. Instead, most studies have compared all voice rehabilitation methods (esophageal speech, tracheoesophageal speech, and electrolarynx) to conclude which is widely preferred in total laryngectomy patients. Finally, another limitation of our research concerns the study of a single rehabilitation technique without comparing its effectiveness with other voice rehabilitation techniques in laryngectomized patients.

## 5. Conclusions

As mentioned above, during the research in the present study, not enough studies were found that give us extensive information exclusively on the QoL of patients who use esophageal speech as a rehabilitation method after total laryngectomy. The data suggest that while ES can bring about some improvements in patient’s communication skills, we should be cautious about patients’ quality of life because of the differences in factors that affect it. Moreover, the psychological and social dimensions of rehabilitation are as important as the functional aspects of voice rehabilitation.

In light of these considerations, future research may focus on larger, more diverse populations and include long-term follow-up to better understand the lasting effects of ES. In addition, a prospective future study concerns comparing the three techniques in terms of effectiveness on overall voice quality, patient preferences, and the quality of life offered by each voice restoration technique. Therefore, future research on evaluating the quality of a patient’s life with esophageal speech in all aspects of personal, social, and professional life, beyond standardized questionnaires, would be interesting. At the same time, the conditions and factors that simplify and help with esophageal speech learning could be investigated to promote it and make it easier to use, as it is the most economical and functional method of rehabilitation because there is no need for constant maintenance and special treatment, as in the case of tracheoesophageal speech and an electrolarynx.

## Figures and Tables

**Table 1:** Critical Appraisal Skills Program (CASP)—(Y: Yes, N: No, CT: Cannot Tell).

(a)						
Qualitative Studies	[35]	[36]	[37]	[5]	[1]	[4]
Was there a clear statement of research aims?	Y	Y	Y	Y	Y	Y
Did the authors look for the right type of papers?	Y	Y	Y	Y	Y	Y
Do you think all the important—relevant studies were included?	Y	Y	Y	CT	N	N
Did the authors do enough to assess the quality of the included studies?	Y	Y	CT	Y	Y	Y
Were the authors clear about the results?	Y	Y	Y	Y	Y	Y
Can the results be applied to the local population?	Y	Y	Y	CT	CT	Y
Were all important outcomes considered?	Y	Y	Y	Y	Y	Y
**(b)**						
Qualitative Studies	[6]	[38]			[39]	
Was there a clear statement of research aims?	Y	Y			Y	
Did the authors look for the right type of papers?	Y	Y			Y	
Do you think all the important—relevant studies were included?	Y	Y			N	
Did the authors do enough to assess the quality of the included studies?	Y	Y			Y	
Were the authors clear about the results?	Y	Y			Y	
Can the results be applied to the local population?	Y	Y			CT	
Were all important outcomes considered?	Y	Y			Y	

**Table 2:** Summary table of articles included in the analysis and their results.

Studies	Title	Sample Size	Groups Consistency	Method	Results
[35]	Voice-Related Quality of Life in Post Laryngectomy Rehabilitation: Tracheoesophageal Fistula’s Wellness.	*n* = 63	Group A: Laryngectomized with TEP Group B: Laryngectomized with ES	VHI	*p* > 0.01 →ES: 38.53 ± 6.62 →TEP:36.24 ± 7.19
				V-QOL	*p* < 0.01 →ES:10.76 ± 2.21 →TEP: 8.73 ± 4.71
[36]	Speech rehabilitation during the first year after total laryngectomy.	*n* = 86 ES	t1: Before total laryngectomy t2: Before or at the very beginning of rehabilitation t3: At the end of inpatient rehabilitation or, in the case of no inpatient rehabilitation, 6 months after total laryngectomy t4: 1 year after total laryngectomy	PLTT	→ES patients: *p* > 0.01 median, PLTT 2.5 vs. 0 →ES objective intelligibility: *p* > 0.01 median, 67.5 vs. 65
[37]	Rehabilitation following total laryngectomy: Oncologic, functional, socio-occupational, and psychological aspects.	*n* = 133	Group A: Healthy population Group B: Laryngectomized population	VHI-30	*p* > 0.01
				EORTC QLQ-C30	*p* > 0.01 →ES: 78.37 ± 7.4 →TEP: 55.9 ± 25.1
				EORTC QLQ-H&N35	No significant data for E.S.
[5]	Voice prosthesis rehabilitation after total laryngectomy: are satisfaction and quality of life maintained over time?	*n* = 42	Group A: Laryngectomized with TEP Group B: Laryngectomized with ES	VHI SF-36	→Reduced social function for ES
				Voice Analysis	
[1]	Quality of life after total laryngectomy: impact of different vocal rehabilitation methods in a middle-income country.	*n* = 22	SCC of larynx, T3/T4,	UW-QOL	→*p* < 0.01 in the speech section TEP →better QoL vs. ES
[4]	Quality of Life in Patients Submitted to Total Laryngectomy.	*n* = 34	Group A: Healthy population Group B: Laryngectomized population	HADS	→ES: Depression: 5.86/Anxiety: 4.29
				P-SECEL	No significant data for E.S.
				EORTC QLQ-C30	No significant data for E.S.
				EORTC QLQ-H&N35	No significant data for E.S.
[6]	Verbal performance of total laryngectomized patients rehabilitated with esophageal speech and tracheoesophageal speech: impacts on patient quality of life.	*n* = 32	Group A: Laryngectomized with TEP Group B: Laryngectomized with ES	VHI	*p* > 0.01 →ES: 37.10 ± 23.02 →TEP: 29 ± 15.87
				VPQ	*p* > 0.01 →ES: 29.2 ± 11.3 →TEP: 23.4 ± 11.9
				V-RQOL	*p* > 0.01 → ES: 10.1 ± 10.8 → TEP: 8.5 ± 2.3
				VOICE ANALYSIS	→F0:*p* < 0.01 ES: 133.09 ± 2.4 TEP: 119 ± 3.3 →NHR: *p* = 0.2 ES: 0.43 ± 0.21 TEP:0.31±0.14 →MPT: *p* = 0.01 ES: 2.02 ± 0.38 TEP: 10.64 ± 0.28
[38]	Comparison of Voice Handicap Index in Patients with Esophageal and Tracheoesophageal Speech after Total Laryngectomy.	*n* = 43	Group A: Laryngectomized with TEP Group B: Laryngectomized with ES	VHI-30	→*p* < 0.01 TEP→29.03 ± 23.479 ES→64.51 ± 21.089
[39]	Satisfaction and Quality of Life in Laryngectomees after Voice Prosthesis Rehabilitation.	*n* = 18	Group A: Laryngectomized with TEP Group B: Laryngectomized with ES Group C: Healthy population	SF-36	→*p* < 0.01 for social-functionality section between A,B

VHI: Voice Handicap Index; V-RQOL: Voice-Related Quality of Life; SF-36: Short-Form Health Survey; EORTC QLQ-C30: European Organization for Research and Treatment of Cancer Quality of Life Questionnaire; EORTC QLQ-H&N35: HEAD & NECK CANCER; UW-QOL: University of Washington Quality of Life; FACT-G: Functional Assessment of Cancer Therapy-General; P-SECEL: Portuguese Self Evaluation of Communication Experiences after Laryngectomy Cancer Questionnaire.

## Data Availability

Not applicable.
